# Impact of an online reference system on the diagnosis of rare or atypical abdominal tumors and lesions

**DOI:** 10.1038/s41598-024-66421-2

**Published:** 2024-07-10

**Authors:** Matthias Michael Woeltjen, Julius Henning Niehoff, Saher Saeed, Alexander Mendorf, Ruth Roggel, Arwed Elias Michael, Alexey Surov, Christoph Moenninghoff, Jan Borggrefe, Jan Robert Kroeger

**Affiliations:** Department of Radiology, Neuroradiology and Nuclear Medicine, Johannes Wesling University Hospital, Ruhr University Bochum, 44801 Bochum, Germany

**Keywords:** Computed tomography, CT, Online reference system, ORS, STATdx, Diagnostic performance, Abdominal tumors, Cancer imaging, Computed tomography

## Abstract

The purpose of the present study is to evaluate whether an online reference system (ORS, STATdx Elsevier, Amsterdam, Netherlands) impacts finding the histologically confirmed diagnosis of rare or atypical abdominal tumors and lesions in radiologic imaging. In total, 101 patients with rare tumor entities or lesions and atypical manifestations of common tumors were enrolled retrospectively. Blinded readings were performed by four radiologists with varying levels of experience, who reported on: (a) correct diagnosis (CD), (b) time needed to find the diagnosis, and (c) diagnostic confidence, initially without followed by the assistance of the ORS. The experienced reader (3 years of experience post-residency, CD 49.5%), as well as the advanced reader with 1 year of experience post-residency (CD 43.6%), and a resident with 5 years of experience (CD 46.5%) made the correct diagnosis more frequently compared to the less experienced reader (CD 25.7%). A significant improvement in making the correct diagnosis was only achieved by the advanced reader, the resident with 5 years of experience (CD with ORS 58.4%; p < 0.001). The advanced reader with 1 year of experience post-residency improved slightly (CD ORS 47.5%). The experienced reader (CD ORS 50.5%) and the less experienced reader (CD ORS 27.7%) did not improve significantly. The overall subjective confidence increased significantly when ORS was used (3.2 ± 0.9 vs. 3.8 ± 0.9; p < 0.001). While the ORS had a positive impact on making the correct diagnosis throughout all readers, it favored radiologists with more clinical experience rather than inexperienced residents. Moreover, the ORS increased the diagnostic confidence of all radiologists significantly. In conclusion, the ORS had no significant impact on the diagnosis of rare or atypical abdominal tumors and lesions except for one reader. The greatest benefit is the increase in diagnostic confidence.

## Introduction

A plethora of rare abdominal tumors and lesions exist. When encountered in clinical routine, these tumors can pose a challenge for the radiologist. However, finding the correct diagnosis is crucial for initiating appropriate therapy. In the past, textbooks and scientific journals reporting on specific tumors and their differential diagnosis served as the main source to identify rare cases. However, given the increasing workloads due to technological advances and an increasing number of abdominal examinations, it is necessary for radiologists to make differential diagnostic considerations and decisions in a reasonable amount of time.

With the advent of the internet in the 1990s, different tools emerged to address this problem and to support radiologists during their clinical routine. In addition, previous studies showed that online applications become increasingly important for training in radiology in general and equally important for assistance in diagnosing rare cases while being on call. A simple Google search was frequently cited as a major source for acquiring fast information^1,2^. Moreover, studies show a positive impact of web-based student teaching formats on the radiological education of medical students and interns^3^ and further promise advantages compared to traditional teaching methods^4^.

Today various online reference systems (ORS) exist. Radiopaedia.org—a free and collaborative website—as well as the commercially available, browser-based ORS named STATdx (Elsevier, Amsterdam, Netherlands), are frequently used by residents and trainees^2,5,6^. Similar ORS, among others, are UpToDate (Wolters Kluwer, Waltham, Massachusetts, USA), Medscape (WebMD LLC., New York, USA), and DiagnosisPro (MedTech USA Inc., Los Angeles, CA, USA)^7–9^. Although ORS are often utilized in clinical practice, to date there have been no studies investigating the diagnostic performance of an ORS.

A similar but further developed reference system is contextflow SEARCH Lung CT (contextflow GmbH, Vienna, Austria) which is a PACS-integrated content-based image retrieval system (CBIRS) to analyze abnormalities of the lung parenchyma in chest CT scans^10^. In CBIRS an algorithm based on artificial intelligence (AI) shows potential diagnoses with associated images and additional educational information after automatically comparing the present CT scan with images in databases^11,12^. Very few clinical and preliminary experimental studies evaluated the diagnostic impact of CBIRS in chest CT scans of the lung parenchyma^13–16^.

STATdx is a browser-based application that is intended to assist radiologists in finding the correct diagnosis. It combines collective clinical experience and knowledge through scientific articles that cover various radiologic topics written by experts in their field of radiology. The subscription-based ORS provides extensive literature and gives overviews of over 4600 diagnoses. Articles are organized by different characteristics, include extensive cross-references, and provide differential diagnoses. Additionally, over 200,000 images of numerous diseases and tumors are included in the online platform, so that radiologists can compare imaging characteristics of their particular case with images on the platform^17,18^.

To our knowledge, there are no other studies dealing with an ORS, so the background is quite limited. Nevertheless, ORS are frequently used in clinical routine and used to provide guidance in diagnosis in unclear cases. The impact that such an ORS has on diagnosis and diagnostic confidence has not yet been evaluated. Given the considerably high cost of subscription-based services, it seems valuable to investigate its utility.

The purpose of the present study is to investigate the impact of the ORS STATdx in correctly diagnosing rare or atypical abdominal tumors and lesions on abdominal CT scans, the time invested to find the correct diagnosis, and the personal diagnostic confidence of the radiologists.

## Material and methods

### Patient population

The study was conducted according to the guidelines of the Declaration of Helsinki and approved by the institutional review board (AZ: 2021-805). Informed consent was waived by the Ethics Committee of the Medical Faculty of the Ruhr University Bochum due to the retrospective study design. All scans were performed for diagnostic use with clinical standard protocols. Patient data were pseudonymized.

In total, 101 patients (54 female and 47 male) with primarily rare abdominal tumor entities or lesions, as well as atypical tumor locations that have been discussed in the institutional interdisciplinary tumor board between 2010 and 2021, were retrospectively included in this study. Some more common lesions were included as well to make diagnostic decision-making more complex. Histology for all was present. Patients were not preselected based on age, gender, weight, or other characteristics.

### CT protocols and image acquisition

All patients underwent a contrast-enhanced CT scan of the abdomen and pelvis in portal-venous phase, additional non-contrast and arterial contrast phases may be present depending on the patient specific indication for the CT scan. Most patients were examined using standard CT scanners at our department; only a few were examined in other regional departments. The scan protocols depended on the individual indication for the CT scan. Only contrast-enhanced CT examinations with at least a portal-venous phase (optional non-contrast and arterial contrast phase) were included in the analysis. All images were reconstructed in the axial view with a slice thickness of 3–5 mm.

The CT images were presented using a standard PACS system (IMPAX 6.7, Agfa Healthcare, Mortsel, Belgium). Readers had access to all available contrast phases of each individual CT examination, but no prior or follow-up exams.

### Reading

The reading was performed independently by 4 blinded radiologists with different levels of experience: Reader 1, a board certified radiologist with 3 years of experience post residency and a specialization in whole-body CT and angiography. Reader 2, a board certified radiologist with 1 year of experience post-residency. After completing his specialist training, he works in the CT team dealing mainly with whole-body CT examinations. Reader 3 and 4 are residents in general radiology with 5 and 2 years of experience, respectively, with a broad spectrum of whole-body CT examinations, various emergency examinations, and cardiac CTs in their training to date. Readers 1 and 3 had several years of experience with the ORS, while readers 2 and 4 had little experience with the ORS. All readers received a dedicated training session and a four-week test period to get familiar with the functionalities of the ORS in the clinical routine.

The readers evaluated the CT scans twice. In the first reading, radiologists made an ad hoc diagnosis, stated up to two differential diagnoses without using the ORS and documented their diagnostic confidence and the required time to establish the diagnosis. The diagnostic confidence was rated using a 5-point Likert scale (1—not confident at all; 2—slightly confident; 3—somewhat confident; 4—fairly confident; 5—completely confident). Immediately afterwards, they were allowed to use the ORS to check or refine their diagnosis. They then documented a diagnosis, up to two differential diagnoses, and their diagnostic confidence. The time taken for the second reading was also recorded.

**Figure 1:**
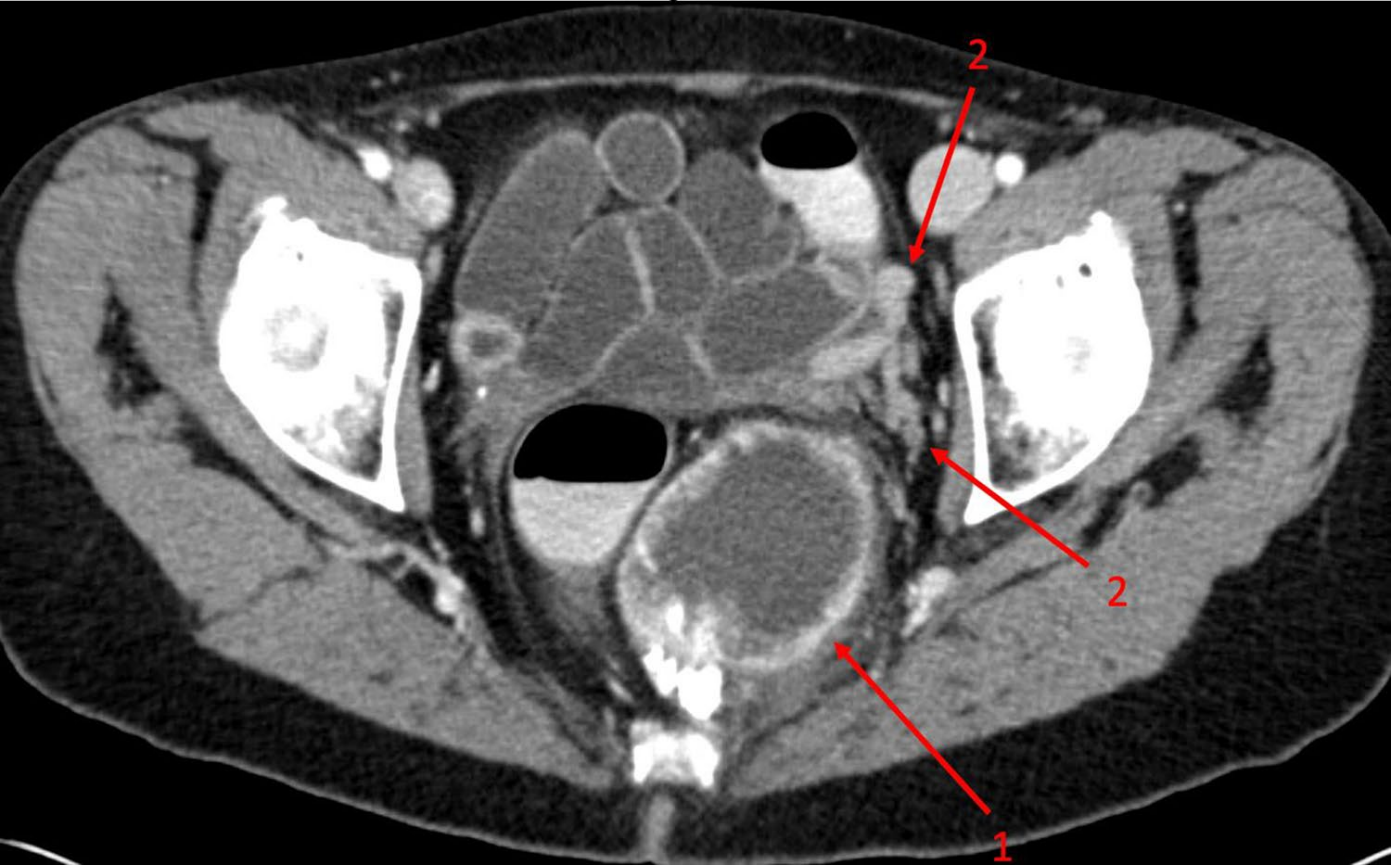
A 62 years old, female patient was admitted to the department of gynaecology with lumbago and paresthesia of the legs. The abdominal portal venous CT scan showed a presacral mass with partial calcifications (1). The tumor exhibited contrast enhancement of the well-defined margin with a more fluid-like necrotic core. Enlarged vessels (2) near the tumor, often seen in solitary fibrous tumors (SFT), were also noted. SFTs typically compress rather than infiltrate surrounding tissues, as seen here by the shifted rectum. Only 1/3 of SFTs are located in the abdomen, and approximately 25% recur locally^19,20^. The diagnosis of an SFT was confirmed histologically after resection.

Readers were previously informed that the goal was to establish the diagnosis as specific as possible. Examples were discussed before the readings. If a reader made no declarations in a case, such as not specifying differential diagnoses, the differential diagnoses were considered incorrect. Exemplary cases are demonstrated in Figs. 1 (solitary fibrous tumor) and 2 (liposarcoma).

**Figure 2:**
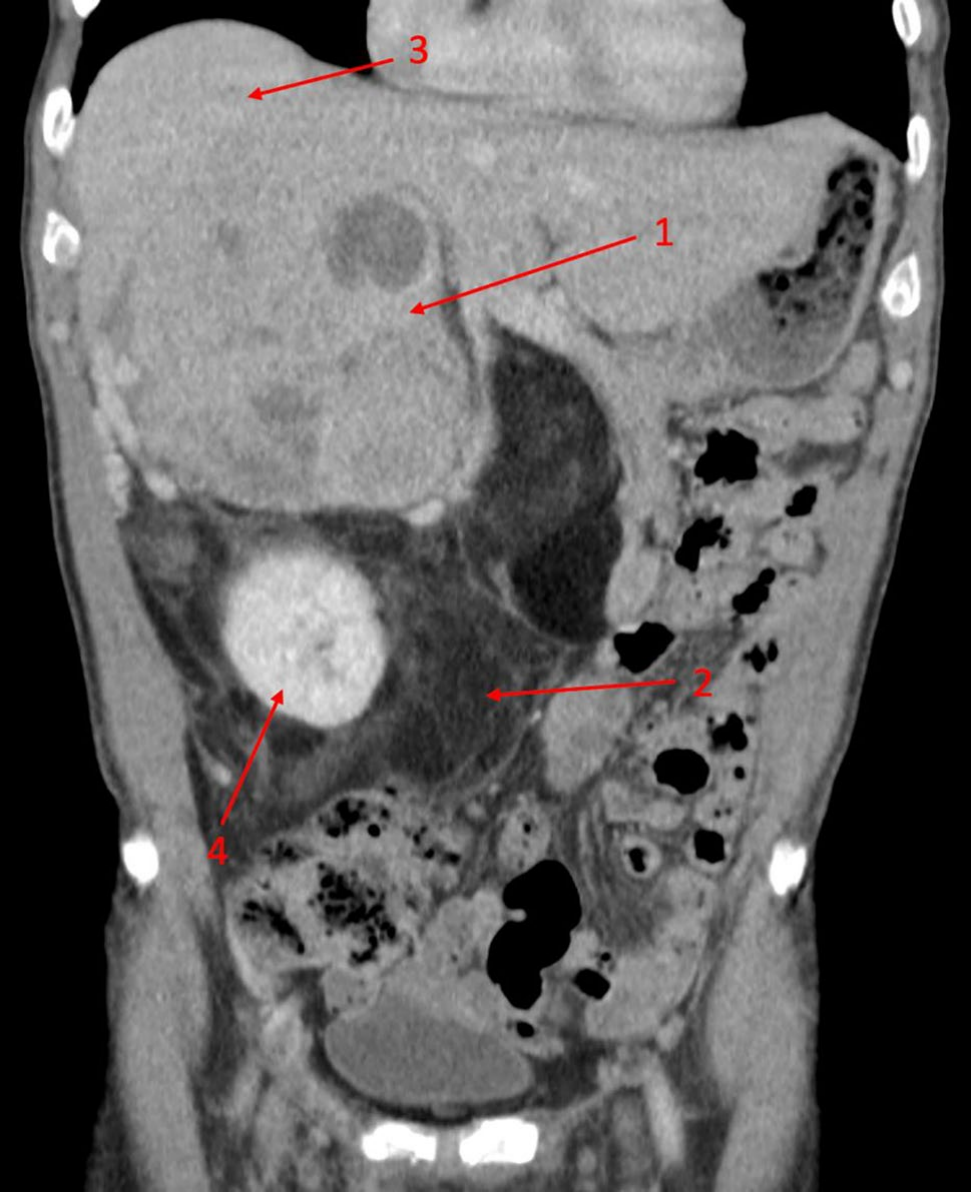
A 74-year-old male patient was referred to visceral surgery for an unclear adrenal mass by his GP. The abdominal portal venous CT scan showed a bimorphic mass. Dedifferentiated liposarcomas typically consist of solid dedifferentiated tumor parts (1) often containing necrosis, and an adjacent lipomatous well-differentiated tumor mass (2). The solid part was adherent to the liver (3), while the lipomatous parts surrounded the kidney (4) and displaced the adjacent intestine. Dedifferentiated liposarcomas typically occur in the retroperitoneal space and mostly arise de novo, while around 10% dedifferentiate from a previous well-differentiated liposarcoma^21^. The initially suspected liposarcoma was later confirmed by the histopathologic report, stating a dedifferentiated liposarcoma with parts of a well-differentiated liposarcoma.

### Statistical analysis

The statistical analysis was performed using established software packages (SPSS Statistics 28, IBM, Armonk, NY, USA/Excel 2016, Microsoft, Redmond, WA, USA/R Core Team (2021); R: A language and environment for statistical computing. R Foundation for Statistical Computing, Vienna, Austria, RStudio Version 1.4.1106).

The ratios of correct diagnosis (CD) and correct differential diagnosis (CDD) were calculated as percentages. Differences between dichotomous variables for CD and CDD were assessed using the McNemar-Bowker test. The Shapiro–Wilk test was utilized to test for normality. Diagnostic confidence and reporting time were not normally distributed, and given as median (Inter-Quartile-Range). The significance of differences in diagnostic confidence was calculated using the Wilcoxon signed-rank test. Statistical significance was assumed for p-values < 0.05.

### Ethics approval and consent to participate

The study was conducted according to the guidelines of the Declaration of Helsinki and approved by the institutional review board (IRB, Ethics Committee of the Faculty of Medicine of the Ruhr University of Bochum, reference number 2021-805). Patient informed consent was waived by the IRB (Ethics Committee of the Faculty of Medicine of the Ruhr University of Bochum) due to the retrospective study design.

## Results

### Cases

Among all included CT examinations (n = 101), 52 different abdominal tumor entities and lesions as well as common tumors with atypical manifestations were identified and histologically confirmed (Table 1).

**Table 1:** Detailed listing of histologically proven rare or atypical abdominal tumor cases that were presented to the readers.

Diagnosis	Number of cases (n = 101, total)
Bile duct adenoma	1
Neuroendocrine tumor of the extrahepatic bile duct	1
Cholangiocarcinoma (intra-/extrahepatic)	6
Neuroendocrine carcinoma of the bile duct	1
Carcinoma of papilla vateri	3
Neuroendocrine carcinoma of papilla vateri	1
Pancreatic adenocarcinoma	7
Pancreatic acinar cell carcinoma	1
Pancreatic neuroendocrine tumor	5
Intraductal papillary mucinous neoplasm (main duct)	4
Intraductal papillary mucinous neoplasm (side branch)	2
Insulinoma	1
Heterotopic pancreatic tissue	1
Pancreatic tubular adenoma	1
Pancreatic pseudocyst	1
Serous cystadenoma of pancreas	2
Pancreatic hypertrophy	1
Solid pseudopapillary tumor (pancreas)	1
Serous microcystic adenoma (pancreas)	1
Hepatocellular carcinoma	6
Macrovesicular steatosis of the liver	1
Hepatic echinococcus cyst	1
Osteoclast-like giant cell tumor of the liver	1
Gallbladder carcinoma	1
Gastrointestinal stromal tumor (GIST) (stomach)	2
Gastrointestinal stromal tumor (GIST) (intestine)	2
Appendix carcinoma	2
Duodenal adenoma	1
Neuroendocrine tumor of the intestine	1
Colon cancer (adenocarcinoma)	1
Duodenal adenocarcinoma (with diverticulum)	2
Ischemic colon ulcer	1
Leiomyosarcoma	5
Liposarcoma	6
Angiosarcoma	3
Extraskeletal myxoid chondrosarcoma	1
Myelolipoma	2
Extrarenal angiolipoma	1
Schwannoma	2
Solitary fibrous tumor	2
Sacral Chordoma	1
Neurinoma	1
Ganglioneuroma	1
Lymphoma	4
Granulosa cell tumor	1
Primitive neuro-ectodermal tumor	1
Pseudomyxoma peritonei	2
Desmoid-type fibromatosis	1
Adrenal cortical carcinoma	1
Primary neuroendocrine tumor of the adrenal gland	1
Malignant pheochromocytoma	1
Primary peritoneal cancer	1

### Correct diagnosis

The percentage of CD with and without the use of the ORS is shown in Table 2.

**Table 2:** Percentage of correct diagnosis (CD) with (+ ORS) and without (ad hoc) the use of the online reference system (ORS).

	CD (ad hoc)	CD (+ ORS)	p-value
Reader 1	49.5%	50.5%	1.000
Reader 2	43.6%	47.5%	0.219
Reader 3	46.5%	58.4%	< 0.001
Reader 4	25.7%	27.7%	0.727

The percentage of CD concurred between readers 1–3 when making ad hoc diagnoses without the use of the ORS, except for reader 4. Among all 4 readers, the most experienced reader, reader 1, attained the highest percentage of CD (49.5%), followed by reader 3 with 46.5% and reader 2 with 43.5% of CD. The lowest percentage of CD was reached by the least experienced reader (reader 4, 25.7%).

By using the ORS, the percentage of CD of all readers increased slightly. Reader 3 improved significantly from 46.5% of CD to 58.4% (p < 0.001). However, the improvements of reader 1, reader 2 and reader 3 were statistically not significant.

### Correct differential diagnosis

The percentage of CDD with and without the use of the ORS is shown in Table 3.

**Table 3:** Percentage of correct differential diagnosis (CDD) with (+ ORS) and without (ad hoc) the use of the online reference system (ORS).

	CDD (ad hoc)	CDD (+ ORS)	p-value
Reader 1	16.8%	15.8%	1.000
Reader 2	14.9%	14.9%	1.000
Reader 3	16.8%	22.8%	0.210
Reader 4	27.7%	29.7%	0.337

Similar to the observations regarding the CD, the percentages of CDD differed between the individual readers when making ad hoc diagnoses without the use of the ORS. However, in terms of CDD, the least experienced reader (reader 4) had the highest percentage of CDD (27.7%), while the other readers achieved lower percentages of CCD (reader 1 16.8%, reader 2 14.9% and reader 3 16.8%).

When using the ORS to find the CDD, reader 3 improved the most (from 16.8 to 22.8% of CDD). Reader 4 improved slightly (from 27.7 to 29.7% of CDD), while the percentage of CDD of reader 1 decreased slightly (from 16.8 to 15.8% of CDD). The percentage of CDD of reader 2 did not change (14.9%). No statistically significant improvement was observed.

### Correct diagnosis or correct differential diagnosis

The percentage of CD or CDD with and without the use of the ORS is shown in Table 4.

**Table 4:** Percentage of correct diagnosis (CD) or correct differential diagnosis (CDD) with (+ ORS) and without (ad hoc) the use of the online reference system (ORS).

	CD or CCD (ad hoc)	CD or CCD (+ ORS)	p-value
Reader 1	60.4%	60.4%	1.000
Reader 2	55.5%	58.4%	0.375
Reader 3	61.4%	76.2%	< 0.001
Reader 4	46.5%	52.5%	0.070

The highest percentage for the CD or CDD without the use of the ORS was achieved by reader 3 with 61.4%, while reader 4 showed the lowest percentage with 46.5%. Reader 3 was the only reader, who improved significantly with the help of the ORS achieving a CD or CDD percentage of 76.2% (p < 0.001). The second-best improvement was achieved by reader 4 (from 46.5% up to 52.5%; p = 0.070). In contrast, the ORS had no impact on the percentage of the CD or CDD of reader 1.

### Diagnostic confidence

**Figure 3:**
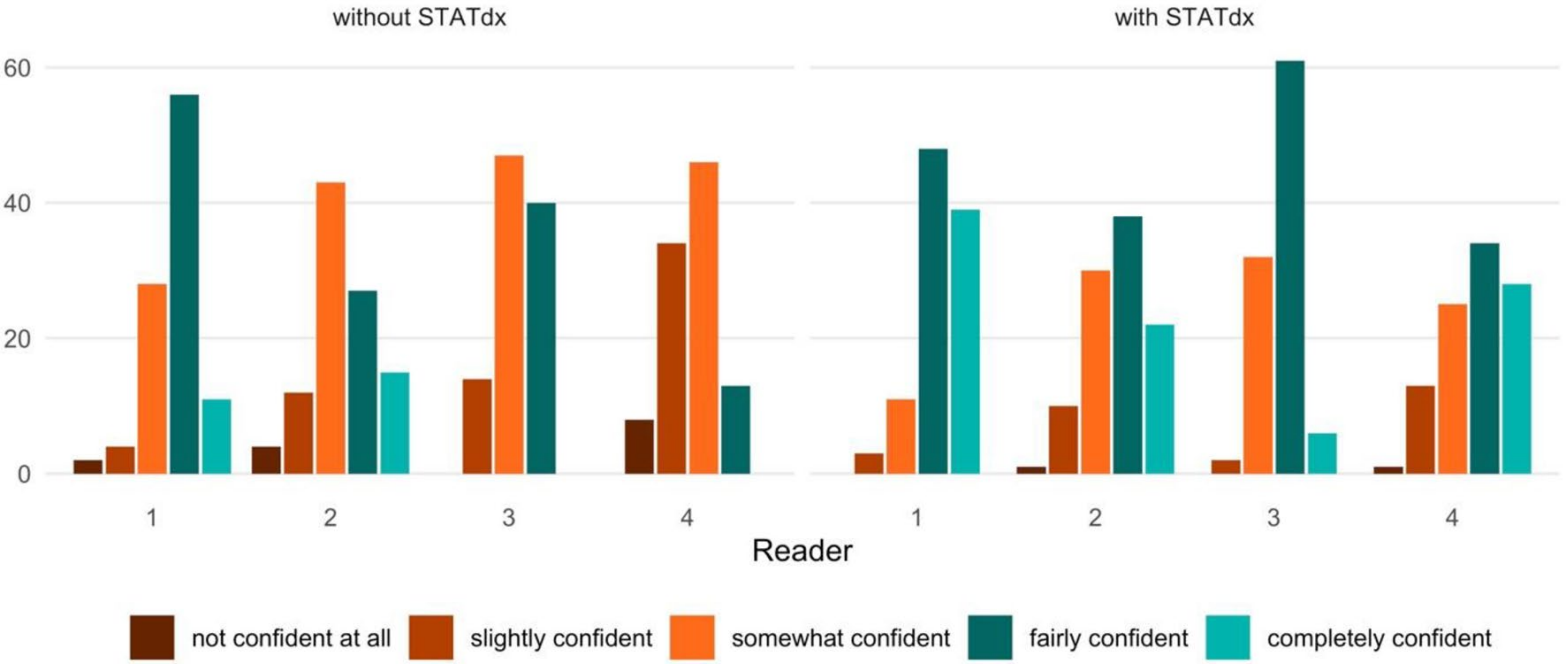
Bar chart showing the diagnostic confidence of the readers (1–4) using a 5-point Likert scale with 1—not confident at all, 2—slightly confident, 3—somewhat confident, 4—fairly confident, and 5—completely confident. The subjective confidence of each reader increased significantly with the use of the online reference system (ORS) STATdx.

The individual diagnostic confidence of the readers is shown in Table 5 and further illustrated in Fig. 3.

**Table 5:** This table shows the diagnostic confidence of the four readers without and with the online reference system (ORS) using a 5-point Likert scale (1—not confident at all; 2—slightly confident; 3—somewhat confident; 4—fairly confident; 5—completely confident) given as median (inter-quartile-range) and their associated p-values.

	Diagnostic confidence (ad hoc)	Diagnostic confidence (+ ORS)	p-value
Reader 1	4 (1)	4 (1)	< 0.001
Reader 2	3 (1)	4 (1)	< 0.001
Reader 3	3 (1)	4 (1)	< 0.001
Reader 4	3 (1)	4 (2)	< 0.001

In total, the median diagnostic confidence as rated by the readers increased significantly from 3(1) (without ORS) to 4 (1) (p < 0.001) when using the ORS.

Without the use of the ORS, reader 1 as the most experienced reader had the highest diagnostic confidence overall with a median of 4 (1) while reader 4 had the lowest diagnostic confidence with a median of 3 (1). The diagnostic confidence of all readers improved significantly when using the ORS (see Table 5 and Fig. 3 for details).

### Invested time

**Figure 4:**
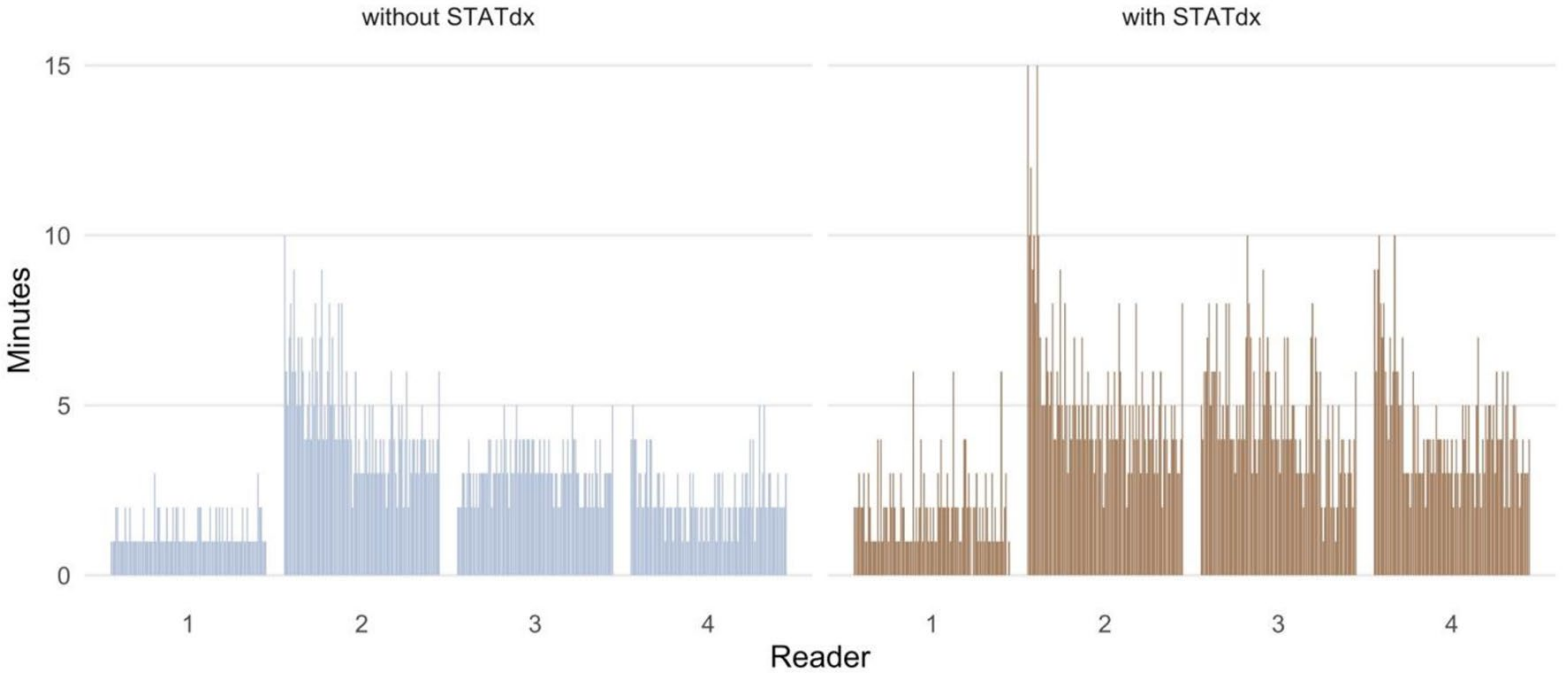
Bar chart showing the expenditure of time of each single reader (1–4) without and with the online reference system (ORS) STATdx in minutes.

The invested time with and without the use of the ORS for each individual reader is shown in Table 6 and displayed in Fig. 4.

**Table 6:** This table displays the expenditure of time of the four readers without and with the online reference system (ORS).

	Time (minutes, ad hoc)	Time (minutes, + ORS)
Reader 1	1.29 ± 0.49	1.96 ± 1.15
Reader 2	4.62 ± 1.69	5.24 ± 2.32
Reader 3	3.11 ± 0.77	4.68 ± 1.80
Reader 4	2.33 ± 0.92	4.30 ± 1.74

Considering all readers, the mean time to make an ad hoc diagnosis was 2.8 ± 1.6 min (= 2 min and 50 s). When using the ORS, the readers needed an additional 4.0 ± 2.2 min (= 4 min 2 s) on average to make a diagnosis.

Among all readers, reader 2 took the most time to find the diagnosis and differential diagnoses (mean 4.62 ± 1.69 min without ORS, mean 5.24 ± 2.32 min with ORS), followed by reader 3. At the same time, reader 1 spent the least amount of time to find the diagnosis (mean 1.29 ± 0.49 min without ORS, mean 1.96 ± 1.15 min with ORS). For the raw data of the reading (CD, CDD, CD or CDD, diagnostic confidence and invested time) see Supplementary Table 1.

## Discussion

This study evaluates the impact of the ORS (STATdx) on diagnostic decision-making. STATdx provides a wide range of clinical and radiological information, among images of various imaging modalities displaying specific diseases, and multiple differential diagnoses. For this study, an extensive set of histologically proven cases with rare or atypical abdominal tumors and lesions, as presented to the tumor board, were identified. The diagnostic performance of four blinded radiologists, both with and without the use of the ORS, as well as their individual diagnostic confidence and the time invested, were examined.

The performance of the radiologists differed in dependence on their radiological experience. The board certified radiologist (reader 1) with 3 years of experience post-residency had the highest percentage of correct diagnoses followed by reader 3 (resident with 5 years of experience) and reader 2 (1 year of experience post-residency) while reader 4 (resident, 2 years of experience) had the lowest percentage of correct diagnoses.

The diagnostic confidence of all readers improved significantly when using the ORS. However, the higher diagnostic confidence did not translate into improved diagnostic performance, as only one reader showed a significant improvement in correct diagnoses when using the ORS. Interestingly, reader 1 needed markedly less time for evaluating the CT scans and utilizing the ORS. In comparison, all other readers, especially readers 2 and 3, took a lot more time, which may have led to a slightly stronger improvement.

In terms of previous experiences with the ORS, it remains unclear whether the greater experience of readers 1 and 3 had an impact on the diagnostic performance. However, the results indicate that there was no major advantage, which may point to the easy usability of the ORS. In contrast to our expectations, the impact on the correct diagnosis for the less experienced reader was very limited. An explanation might be that experience is needed to point the user in the right direction of a disease. Clinical inexperienced users might struggle with the vast number of possible diagnoses if they cannot assign an unknown tumor to a specific tissue type. Consequently, further differentiation becomes difficult to achieve.

To the best of our knowledge, no other study has investigated the impact of STATdx or any other ORS on the diagnostic performance of radiologists. Nevertheless, a few studies on similar topics exist. Roehrich et al. evaluated the content-based image retrieval system (CBIRS) contextflow SEARCH Lung CT (contextflow GmbH, Vienna, Austria), which is a PACS integrated application based on AI algorithms that suggests diagnoses and provides images and additional information. Although they observed a trend toward improved overall diagnostic accuracy, the improvements did not reach statistical significance^16^. In a preliminary study, Aisen et al. evaluated the impact of a newly developed CBIRS. Eleven radiologists with different levels of experience analyzed 29 images with different lung diseases without CBIRS and one week later with the assistance of their CBIRS. The diagnostic accuracy improved significantly from 29 to 62%^15^. Although CBIRS and ORS employ different approaches, both systems proved to have a positive impact on the diagnostic performance of radiologists.

While only one reader achieved a statistically significant short-term improvement, long-term improvement may be implied since all readers recognized the ORS as a helpful tool, as reflected by the increased diagnostic confidence. However, this increased confidence in making a diagnosis could also potentially reinforce errors to some extent.

Time efficiency and short-term improvements may be enhanced through different means. The ORS could be extended to a CBIRS application that presents selected images with possible diagnoses and offers additional information on differential diagnoses^12,16,22^. An example of such a CBIRS application is the aforementioned contextflow^10,16^.

Our study has certain limitations. The monocentric study design as well as the limited number of cases. The small number of readers and their varying levels of experience may not reflect the full potential for short-term improvement. In particular, a reader with many years of experience could provide additional benefits and better distribution in reader experience. A larger study with various readers from multiple radiological institutions is needed to explore these limitations further. Another limitation is the possibility of a selection bias that further affects the results. Cases were preselected on the basis that the diagnosis was deemed rare or atypical. However, no they were not selected regarding the possible impact of an ORS. Thus, in many cases the ORS probably was not able to provide meaningful impact on the diagnosis. In this study, no power calculation was performed to determine the number of cases needed. We think it is not possible to make a good assumption as it is the first study that examines the diagnostic performance of an ORS. The study is only of limited clinical relevance, as there is no direct benefit for the patient. Most tumors are classified bioptically. A precise radiological diagnosis cannot replace this. However, this study is primarily concerned with the ORS STATdx itself and its influence on diagnosis regardless of clinical relevance. It is possible that the influence of the ORS is more pronounced in a different set of cases.

In conclusion, the ORS marginally improved the diagnostic performance of the readers, but no statistically significant impact on diagnostic decision-making was observed, except for one reader. However, diagnostic confidence increased significantly throughout all readers. Differences in finding the correct diagnoses and differential diagnoses between the readers may be associated with their radiological experience and the time invested in each case. Contrary to our expectations, the ORS had a greater impact on the correct diagnosis of experienced readers rather than less experienced readers. Additional improvement can be expected with longer use of the ORS in the clinical routine. However, the use of the ORS significantly increased the time needed to assess a case, limiting its use in the clinical routine. The greatest benefit may be the significantly increased diagnostic confidence.

### Supplementary Information

Supplementary Table 1.

## Data Availability

The data are available from the corresponding authors with reasonable requests.

## Abbreviations

ORS: Online reference system
CD: Correct diagnosis (CD)
CDD: Correct differential diagnosis
CT: Computed tomography
CBIRS: Content-based image retrieval system
AI: Artificial intelligence
SFT: Solitary fibrous tumor
